# Treatment performance comparison between regular O_3_–BAC and O_3_–BAC with rear sand filtration: verification in a full-scale study

**DOI:** 10.1186/s12302-019-0229-6

**Published:** 2019-07-12

**Authors:** Kai Yang, Jianwei Yu, Qingyuan Guo, Chunmiao Wang, Ping Xia, Y. Jeffrey Yang, Min Yang

**Affiliations:** 1 Key Laboratory of Drinking Water Science and Technology, Research Center for Eco-Environmental Sciences, Beijing 100085, China.; 2 University of the Chinese Academy of Sciences, Beijing 100019, China.; 3 Yancheng Institute of Technology, Yancheng 224051, China.; 4 Shanghai National Engineering Research Center of Urban Water Resources Co., Ltd., Shanghai 200082, China.; 5 US Environmental Protection Agency, Office of Research and Development, Cincinnati, OH 45286, USA.

**Keywords:** Sand–O_3_–BAC, O_3_–BAC–sand, Sand filter interception, Drinking water treatment

## Abstract

**Background:**

To improve the microbial safety of drinking water, an arrangement of O_3_–BAC with rear sand filtration (O_3_–BAC–sand) has been proposed. In this study, efforts were devoted to evaluate the benefits and drawbacks of O_3_–BAC–sand in a full-scale water treatment plant. The performance of the two configurations was compared in terms of particles, turbidity, COD_Mn_ and typical odorants and pesticides.

**Results:**

The O_3_–BAC–sand yielded lower turbidity but higher COD_Mn_ (by approximately 7%) in the finished water than regular O_3_–BAC (sand–O_3_–BAC). Both systems removed odors in raw water; however, sand–O_3_–BAC was more effective on septic and musty odorants. The total pesticide removals by sand–O_3_–BAC and O_3_–BAC–sand were 78% and 72%, respectively; though the latter had shorter activated carbon durable years.

**Conclusion:**

The re-location of the sand filter would sacrifice the BAC efficiency in removals of organic matter and micropollutants. This tradeoff is a result of the loss of the particulate organic matter removal by sand filters, because locating the sand filter behind BAC causes particle load increase on BAC; some measures of enhanced coagulation should be suggested to improve the turbidity and particle removal. The study will be helpful for improvement of the O_3_–BAC process in drinking water treatment.

## Background

Ozonation integrated with biological activated carbon (O_3_–BAC) is adopted more and more often for drinking water purification because of its effectiveness in removing disinfection byproduct precursor sand synthetic organic chemicals [1]. In the treatment train, it is common practice that the O_3_–BAC unit is placed after sand filtration (regular O_3_–BAC: sand–O_3_–BAC) [2], and the presence of some genera might play important roles in the biodegradation taking place in the BAC filter [3]. During the operation, BAC filter would provide a suitable condition for the growth of aquatic micro-animals, which could form a microorganism–protozoa–macroinvertebrate biological chain [4]. Moreover, particles harboring bacteria, particularly some chlorine-resistant pathogens like *Legionella* and *Chromobacterium* [5], could possibly penetrate the BAC bed, and the microorganism could also get through the BAC filter, which would have impact on the drinking water quality. This has been reported in some O_3_–BAC treatment plant, especially in the south subtropical region in China [6], giving rise to biological safety concerns of the drinking water. One option is O_3_–BAC with rear sand filtration (O_3_–BAC–sand), which provides a barrier for reducing the biological risk [7] and has been reported in previous studies [8, 9]. And the O_3_–BAC–sand process has been applied in some water treatments in China such as Shanghai [10], Fuzhou [11], Jinan [12] and Jiaxing [13].

Sand filtration is one key step in drinking water treatment to remove particles from raw water [6, 14], and it is also known to be inefficient for removing hydrophilic pollutants, because hydrophilic compounds do not easily adsorb into sand filter beds, whereas hydrophobic compounds with log*Kow* > 2.5 tend to adsorb onto particles, resulting in higher removal efficiencies (> 80%) during sand filtration [15, 16]. The filter media intercept and remove particles harboring organic compounds, as well as organic particles like algae escaping from the upstream sedimentation unit [16, 17]. Consequently, post-sedimentation particulate organic matters may enter the ozonation unit when the sand filter is located downstream of the BAC filter. Furthermore, particulate organic matter can be transformed into dissolved organic carbon (DOC) during the ozonation process [18], and thus increase mass load on the BAC filter. These treatment tradeoffs should be evaluated to determine the benefits and drawbacks of the rear sand filtration for a given drinking water treatment plant.

In this paper, the treatment performance for the rear sand filter arrangement is investigated and compared through experimental studies in a full-scale drinking water treatment plant with two parallel configurations (sand–O_3_–BAC and O_3_–BAC–sand). Treatment performance was compared in the removal of chemical oxygen demand by KMnO_4_ titration (COD_Mn_), the only parameter of comprehensive organic material concentration in the Chinese drinking water standard, and particles, turbidity, typical odorants as well as some typical pesticides.

## Materials and methods

### Comparative treatment analysis

The water treatment plant of 7 × 10^5^ m^3^/days capacity in Shanghai, China has two parallel treatment systems: a sand–O_3_–BAC process and a modified treatment train equipped with a rear sand filter (O_3_–BAC–sand). The sand–O_3_–BAC system, of 4 × 10^5^ m^3^/days production capacity, consists of pre-ozonation, coagulation, sedimentation, sand filtration, post-ozonation and BAC in succession. The O_3_–BAC–sand system, of 3 × 10^5^ m^3^/days capacity, varies in the location of sand filtration: pre-ozonation, coagulation, sedimentation, post-ozonation, BAC and sand filtration in succession. The sand–O_3_–BAC and O_3_–BAC–sand systems have operated for 47 and 33 months, respectively.

During the four-month study period, typical operation parameters were aluminum sulfate at 40 mg/L (8.0 mg/L as Al_2_O_3_ concentration) dose, 105-min sedimentation time, ozone dose of 0.5 mg/L for pre-ozonation (5 min) and 1.0 mg/L for post-ozonation (15 min), sand filtration velocity of 7.9 m/h, the empty bed contact time and filtration velocity of the BAC filter were 16 min and 8.3 m/h, respectively.

The unit processing and treatment efficiency were examined for samples collected at the raw water intake and the effluents from the sedimentation, sand filtration, post-ozonation and BAC units from May to August in 2015. Analyte included turbidity, particle counts and COD_Mn_, odorant and pesticide compounds. For the determination of turbidity, particle counts and COD_Mn_, a 1-L water sample was taken once or twice every week, and analyzed right after sampling. The samples for odorant determination were taken thrice in May and twice every month, stored in a refrigerator after filtration with a glass fiber filter and then taken back to the laboratory for the analyses. The samples for pesticide determination were taken once every month from the systems.

### Sample preparation and analysis

The COM_Mn_, particle size, odor compounds, and pesticides were analyzed for the collected samples. All reagents used in the experiment were of guaranteed reagent grade, and all stock solutions were prepared with MilliQ water (Millipore). COD_Mn_ was measured on a HACH Model DR2800 spectrophotometer (HACH, USA). Soluble COD_Mn_ (SCOD_Mn_) was obtained by measuring the samples after 0.45-μm membrane (GF/F, Whatman, UK) filtration. Insoluble COD_Mn_ (inSCOD_Mn_) equals Total COD_Mn_ (TCOD_Mn_) subtracting SCOD_Mn_.

Concentrations of 25 pesticides (Table 1) were measured using GC/MS according to Yu [19]. The flavor profile analysis (FPA) method was used to characterize the odors according to the Standard Methods for the Examination of Water and Wastewater [20]. Typical septic (dimethyl disulfide (DMDS), dimethyl trisulfide, bis(2-chloro-1-methylethyl) ether and musty (2-methylisoborneol (2-MIB)) odorants were determined by two-dimensional gas chromatography with time-of-flight mass spectrometry (GC × GC–TOFMS) using the methods in [21].

Finally, particle size distribution was characterized on a Hybrid Particle Counter ZVL (Fuji Electric, Japan), in grab sample mode. The method measures both turbidity and particle count in 9 size ranges (1–2 µm, 2–3 µm, 3–5 µm, 5–7 µm, 7–10 µm, 10–15 µm, 15–20 µm, 20–30 µm, > 30 µm).

## Results and discussion

During the study period, the raw water turbidity ranged from 18.8 to 46.0 NTU, COD_Mn_ from 4.50 to 6.84 mg/L, counts for particles of 1–2 µm, 2–5 µm, 5–15 µm and > 15 µm from 268,836.00 to 731,408.25, from 32,278.64 to 294,281.25, from 18,726.58 to 47,688.50, and from 61.25 to 1783.08,respectively. It is also known for its septic/swampy odor occurrence and the presence of a variety of micropollutants because of pollution in source water [22].

### Turbidity and particles

**Fig. 1:**
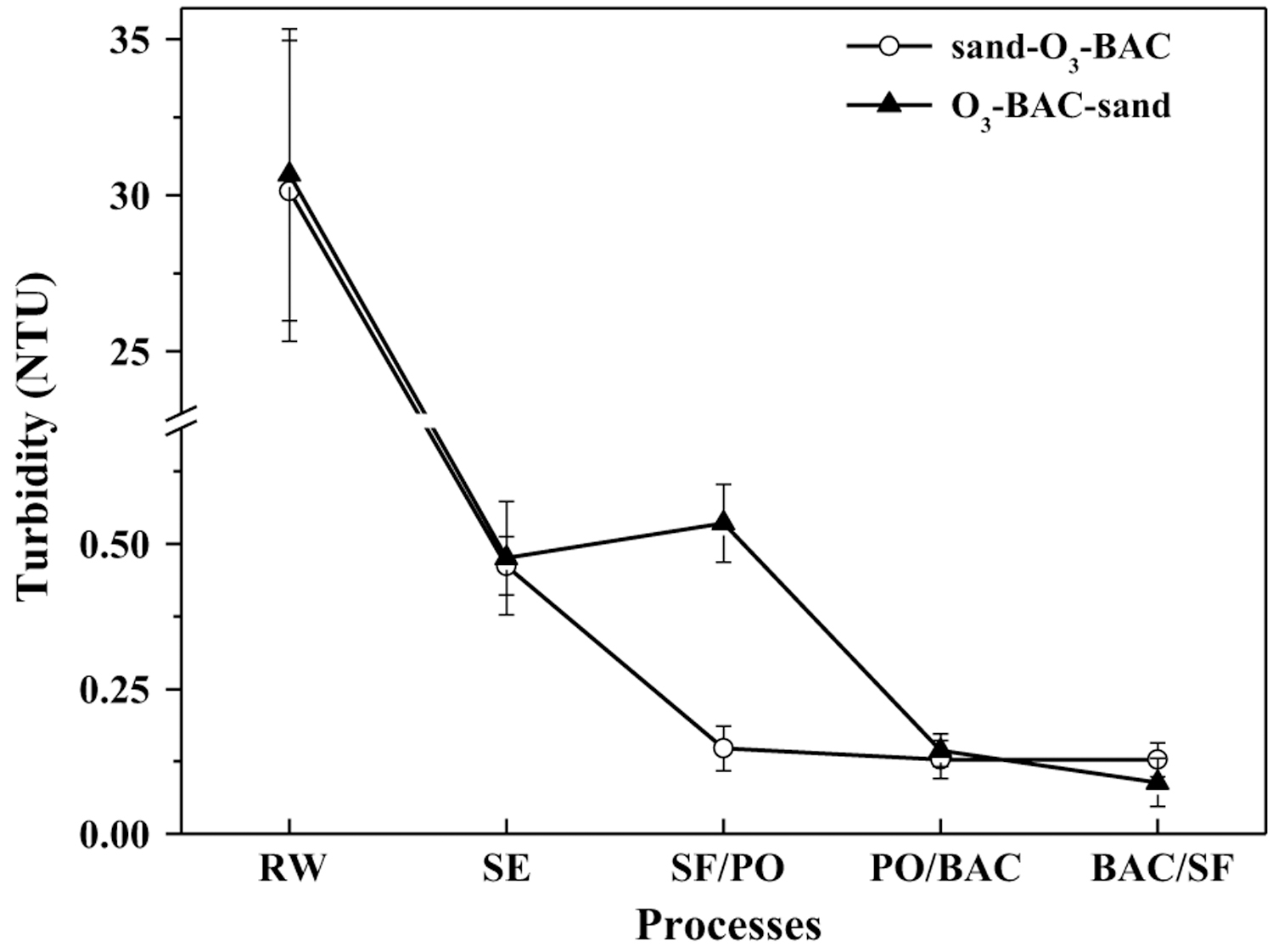
Changes of turbidity along the treatment trains. *RW* raw water; *sand–O*_*3*_*–BAC* regular O_3_–BAC; *O*_*3*_*–BAC–sand* O_3_–BAC with rear sand filtration; *SE* sedimentation; *SF* sand filtration; *PO* post-ozonation. Operational conditions: pre-ozone dose 0.5 mg/L; post-ozone dose 1.5 mg/L. The error bars represent mean ± S.D

**Fig. 2:**
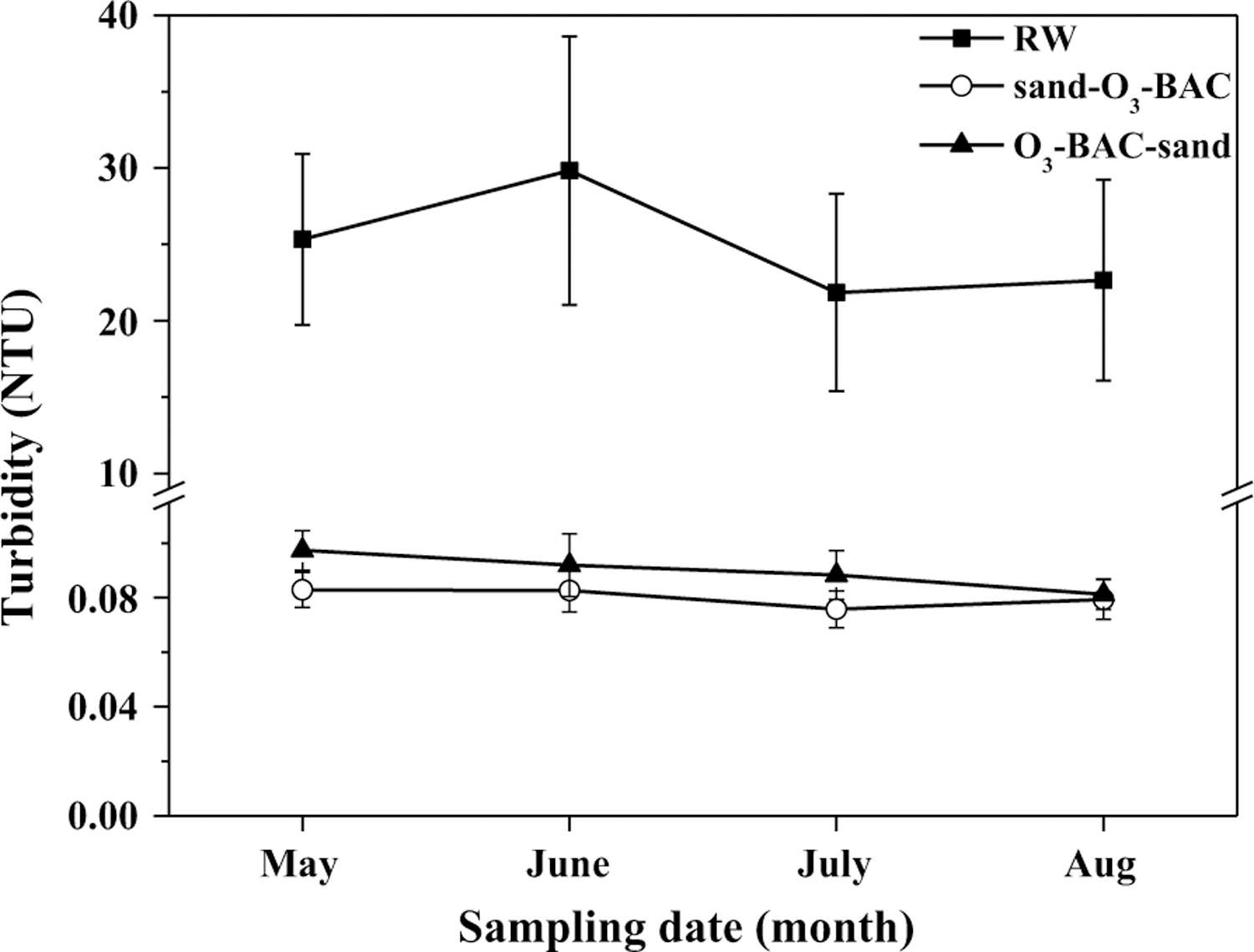
Month average turbidity results of the finished water. *RW* raw water; *sand–O*_*3*_*–BAC*: regular O_3_–BAC; *O*_*3*_*–BAC–sand* O_3_–BAC with rear sand filtration (Based on the day-to-day monitoring data from drinking water treatment plant; sampling date: from May to Aug, 2014). The error bars represent mean ± S.D

**Fig. 3:**
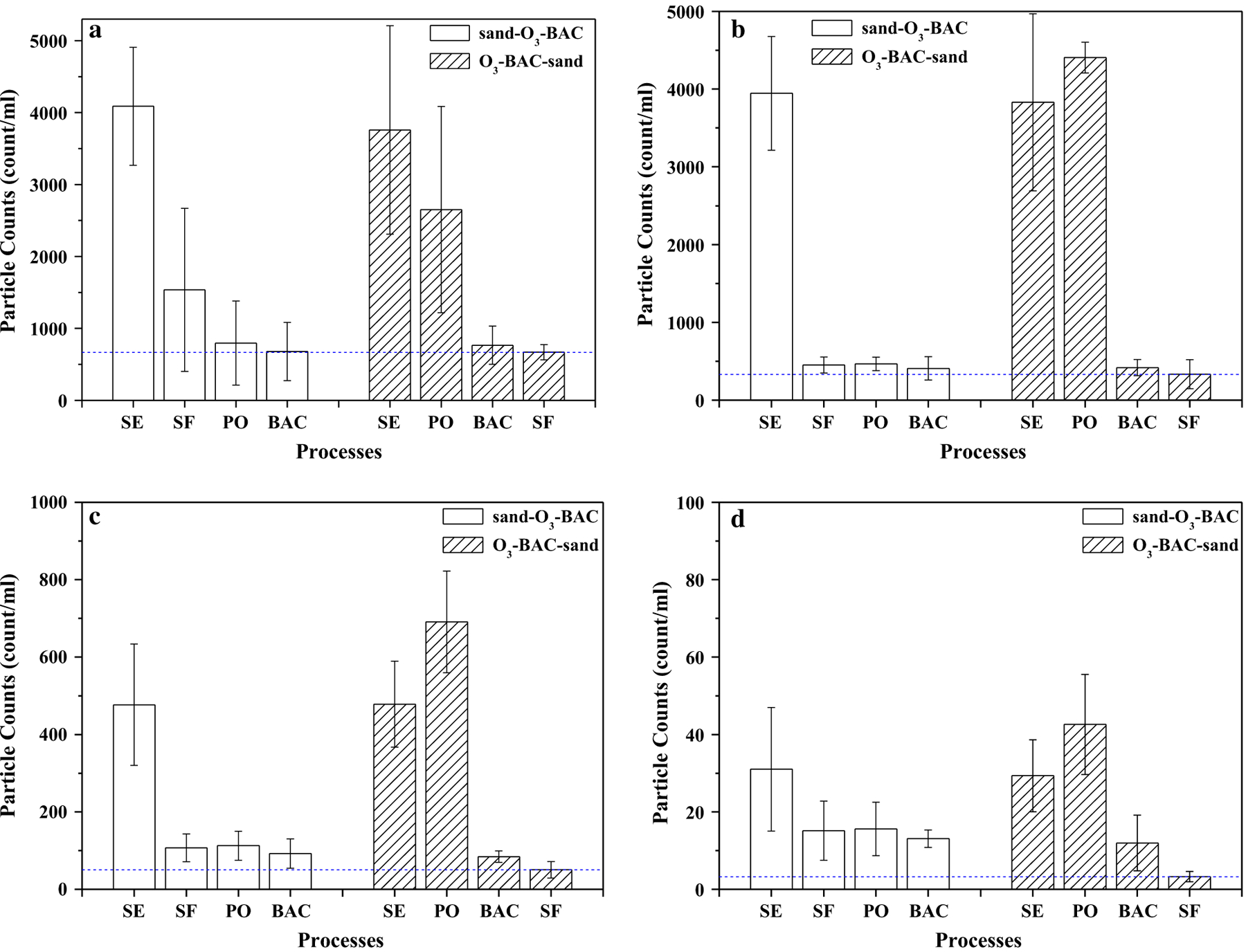
Changes of particle counts along the treatment trains. **a** 1–2 μm; **b** 2–5 μm; **c** 5–15 μm; **d** > 15 μm. *sand–O_3_–BAC* regular O_3_–BAC; *O_3_–BAC–sand* O_3_–BAC with rear sand filtration; *SE* sedimentation; *SF* sand filtration; *PO* post-ozonation. The dotted line is the particle counts value of sand filtration effluent in the rear sand filtration process. The error bars represent mean ± S.D

Figure 1 shows the changes of average turbidity along the treatment processes in the two systems. Generally, the finished water in the rear sand filtration system has lower turbidity; in average, 0.12 NTU in sand–O_3_–BAC vs. 0.04 NTU in O_3_–BAC–sand (*p* = 0.020). The day-to-day monitoring data of the finished water also exhibited similar results (Fig. 2). For the sand–O_3_–BAC system, the turbidity removal primarily occurred in the sand filtration. Turbidity removal in the O_3_–BAC–sand process occurred in both the BAC and sand filters. A slight increase of turbidity after ozonation (Fig. 1) may be the result of fine particle aggregation and subsequent breakup into small sizes, a process reported in [23, 24]. Besides, oxidation of iron and manganese, which can produce some insoluble inorganics like goethite, magnetite and MnO_2_, has also been reported to be responsible for turbidity increase after ozonation [25]. Changes of particle counts across the treatment train were examined in particle size ranges (Fig. 3). Clearly, in sand–O_3_–BAC system, sand filtration removed particles over all size ranges, and ozonation was effective in removing small particles of 1–2 µm. In O_3_–BAC–sand system, significant decrease in number concentration of small particles (1–2 µm) following ozonation was in contrast to an increase for the larger particles (> 2 µm). This observation apparently agrees with previous reports [3, 26] that ozonation destabilized small particles and aggregated to form larger particles. Dissolution of some small organic particles (1–2 µm) as a result by ozonation, on the other hand, may also be possible. Interesting to note, largest particle removal occurred in the BAC unit (Fig. 3) potentially as a result of its physical filtration and biodegradation functions. The average counts for particles of 2–5 µm, 5–15 µm and > 15 µm in the finished water of O_3_–BAC–sand system were 331.45, 50.06 and 3.14 count/mL, respectively, in comparison with 460.4, 109.8, and 14.0 count/mL, respectively, in sand–O_3_–BAC system.

### COD_Mn_

**Fig. 4:**
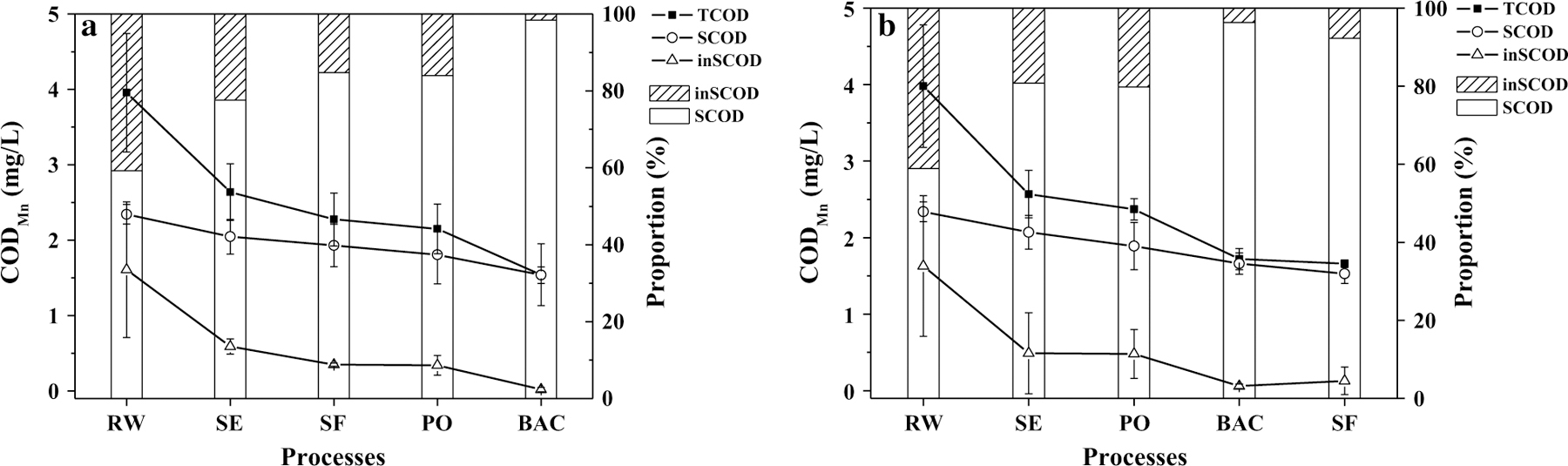
Changes of CODMn concentrations along the treatment trains. **a** sand–O_3_–BAC, **b** O_3_–BAC–sand. *RW* raw water; *SE* sedimentation; *SF* sand filtration; *PO* post-ozonation. The error bars represent mean ± S.D

**Fig. 5:**
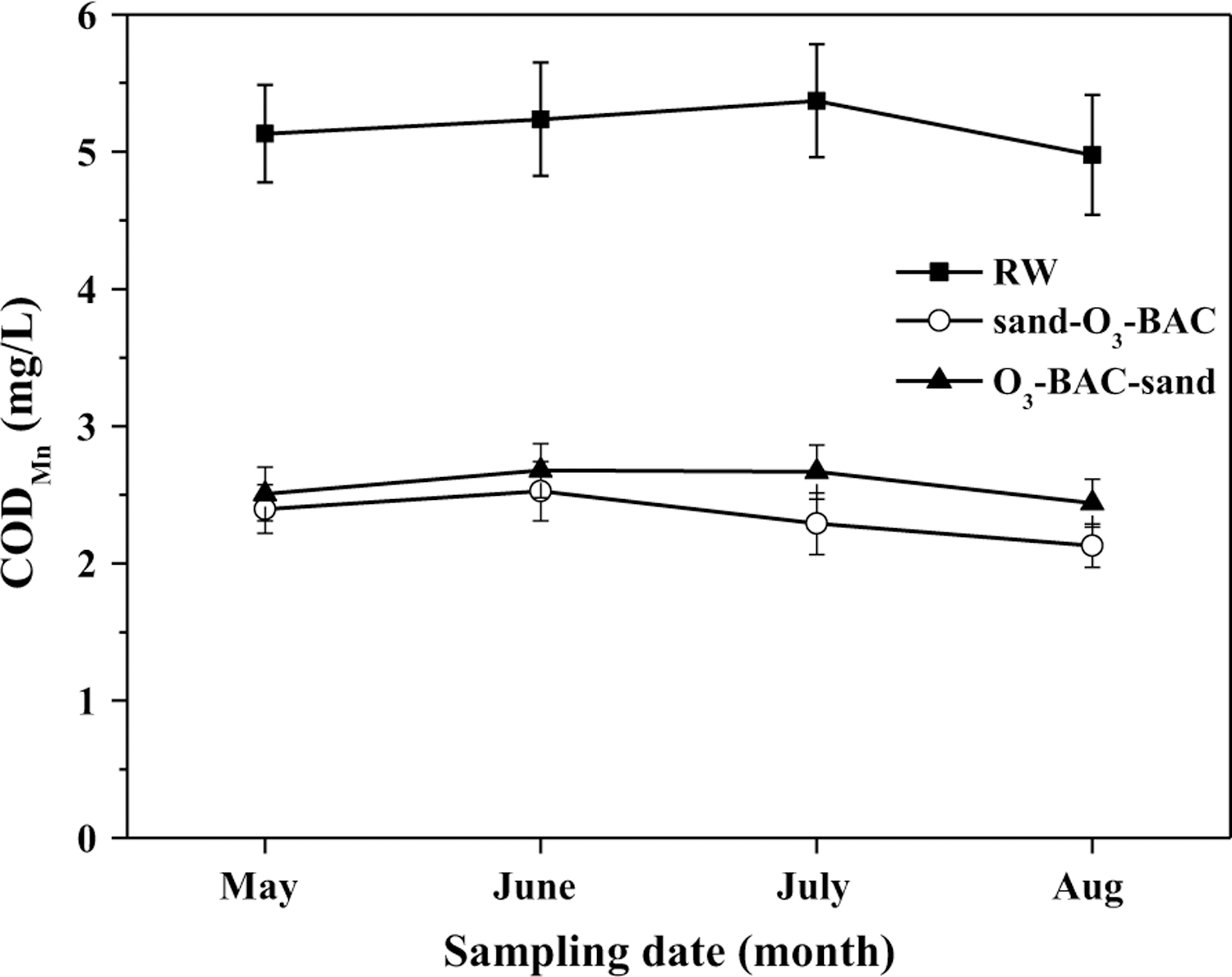
Month average of day-to-day total COD_Mn_ monitoring data of the finished water. *RW* raw water; *sand–O*_*3*_*–BAC*: regular O_3_–BAC; *O_3_–BAC–sand* O_3_–BAC with rear sand filtration (Based on the day-to-day monitoring data from drinking water treatment plant; sampling date: from May to Aug, 2014). The error bars represent mean ± S.D

The changes in COD_Mn_ along the treatment processes are shown in Fig. 4 and details are listed in Table 2. The COD_Mn_ value of finished water in sand–O_3_–BAC system was approximately 7% lower than that in O_3_–BAC-sand system. The statistically significant difference shows the better COD_Mn_ removal in sand–O_3_–BAC. Similarly, day-to-day monitoring data of the finished water (Fig. 5) showed consistently lower COD_Mn_ in the finished water of sand–O_3_–BAC by an average of 0.12 mg/L.

The main COD_Mn_ removal in sand–O_3_–BAC system occurred in coagulation–sedimentation, BAC and sand filtration (Table 3). Post-ozonation treatment only contributed a small fraction of the overall COD_Mn_ removal. Here, ozonation functions to transfer organic molecules into smaller ones, such as acetic acid, aldehydes, and ketones, which could be assimilated into biomass in the following BAC filter [27, 28]. By contrast, in O_3_–BAC–sand, COD_Mn_ was largely removed by coagulation–sedimentation and BAC, while the COD_Mn_ removal by sand filtration was negligible. It is known that sand filtration is primarily used for the removal of particles downstream of the sedimentation process unit, in which organic particles like algae and small flocs harboring organic compounds are trapped resulting in the removal of inSCOD_Mn_ [29]. Without the interception by sand filters, these particles would be oxidized directly by ozone and some would be trapped by the BAC filters.

We consider that the ozone consumption for inSCOD_Mn_ removal may have affected other unit performance such as the efficiency of bio-refractory NOM transformation by ozonation. Without the interception by sand filters in the O_3_–BAC–sand system, these particles would be oxidized directly by ozone and some would be trapped by the BAC filters. The consumption of ozone by inSCOD_Mn_ could affect the removal efficiency of the bio-refractory organic compounds, including NOM, by ozonation. Moreover, BAC has actually taken on the filtration function in O_3_–BAC–sand system; thus, the particle and inSCOD_Mn_ loading was higher than in sand–O_3_–BAC system. The increase in particle and inSCOD_Mn_ loading to the BAC filter may change the BAC filter surfaces resulting in lower efficiency for SCOD_Mn_ biodegradation. Filter clogging also necessitates the increase in backwashing frequency, for example from once per 3 or 4 days to once a day in previous research [30], and from 102 to 72 h in this study, which would affect not only the biodegradation performance, but also the service life of BAC due to the increased wearing down of activated carbon [31].

For these reasons, the adoption of O_3_–BAC with rear sand filtration in the treatment would encounter two drawbacks in COD_Mn_ removal: competition with soluble COD_Mn_ ( SCOD_Mn_) for ozone by i nSCOD_Mn_, and secondly, interference by accumulating particles in BAC to its biodegradation functions. The net result is lower COD_Mn_ removal efficiency of the rear sand filtration process (Fig. 4). To address the negative impact, Han et al. [7] suggested that up-flow BAC filtration may increase the COD_Mn_ removal by, a possibility that warrants further investigations.

### Typical odorants

**Fig. 6:**
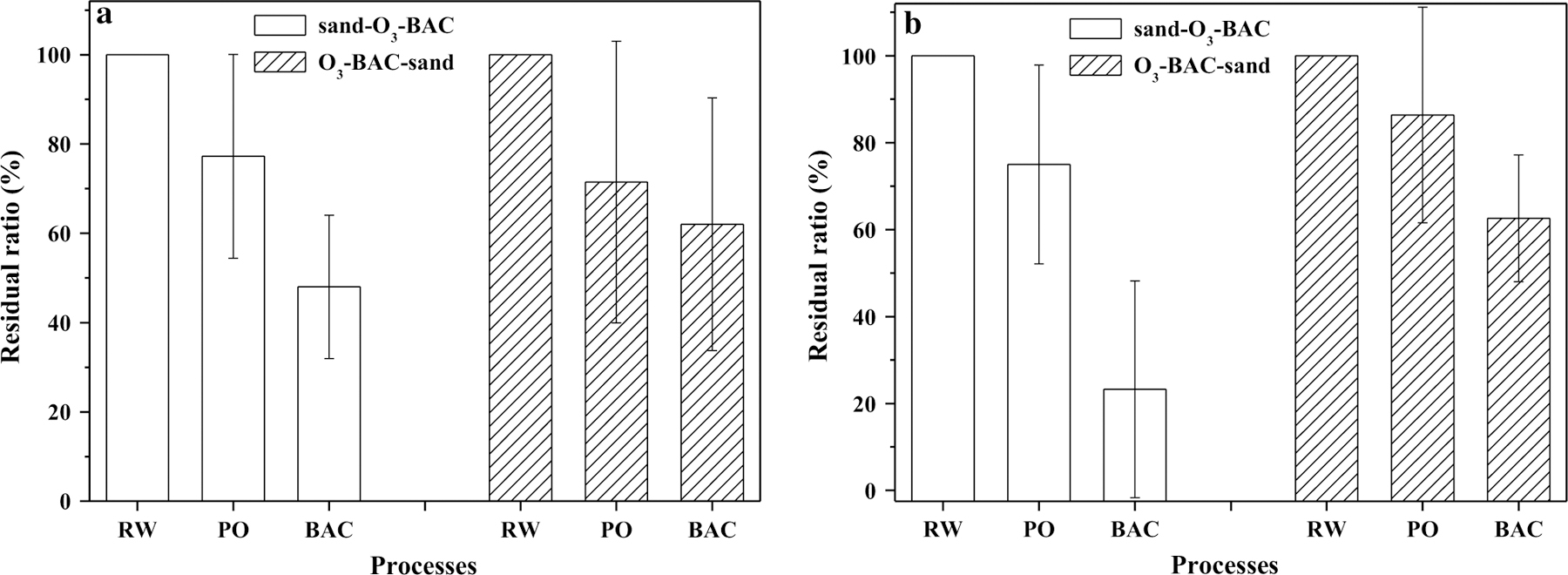
Changes of odorant concentrations along the treatment trains. **a** Sum of dimethyl disulfide, dimethyl trisulfide and bis (2-chloro-1-methylethyl) ether, **b** MIB. The error bars represent mean ± S.D

Over the four-month period, the FPA intensity in raw water was 5–8 for the septic odor and 4–6 for the musty odor, indicating the moderate to strong odor characteristics. In general, an FPA level of lower 3 was thought to be acceptable for drinking water [32]. Our previous study [22] has shown the periodic occurrence of musty odor and long-term septic odor in the Huangpu River source water. The removal of the main odors and odorants by O_3_ and BAC is shown in Fig. 6. While the finished water from the two systems was odorless, the odorant removal performance was different. The raw water contained 26.7–72.4 ng/L septic odorants (total concentration of dimethyl disulfide, dimethyl trisulfide and bis (2-chloro1-methylethyl) ether) and 9.2–15.9 ng/L musty odorant (MIB). Overall, BAC filters in sand–O_3_–BAC system removed more septic and musty odorants than those in O_3_–BAC–sand system. The lower odorant removal efficiency in O_3_–BAC–sand system is likely attributed to the occupation of BAC surfaces by particles and the increased frequency of backwashing. The increase of backwashing frequency could decrease the biomass amount in the BAC filter,which might lead to unstable reactor performance [33]. The biomass and activity on the BAC filter might also have an impact on the odorant removal [34], which needs further investigation.

### Pesticides

As shown in Table 4, among the 25 investigated, eight pesticides [machette, hexachlorobenzene (HCB), 2,2bis (p-Chlorophenyl)-1,1,1-trichloroethane (p,p’-DDT), dimethoate, fenobucarb, dichlorvos, acetochlor, and atrazine] were detected in the raw water. The concentration ranges from 3.94 to 646.81 ng/L, similar to those of our previous study [35]. As indicated in Table 4, the removal of pesticides was mainly contributed by ozonation (19.41%) and BAC (32.09%) in sand–O_3_–BAC, while the removal was by BAC in O_3_–BAC–sand system (42.58%). The total removals for sand–O_3_–BAC and O _3_–BAC–sand were 78.43% and 70.03%, respectively.

The lower pesticide removal by ozonation in O_3_–BAC–sand might be attributed to the competition for ozone by the particulate organic matters (inSCOD_Mn_). Because of sand filtration upstream of ozonation, sand–O_3_–BAC benefited from the sand filter in removal of pesticides (14.70%) that are probably adsorbed in the particles. However, for both systems, BAC showed a relatively high removal rate for the pesticides that are mostly biorefractory, mainly because of the carbon adsorption [36, 37]. Relatively, higher pesticide removal was observed in sand–O_3_–BAC than O_3_–BAC–sand system, suggesting that the process offers better efficiency in the rem oval of micropollutants like pesticides. The difference in pesticide removal can be also potentially attributable to the performance of BAC unit process. Prior to the comparative treatability study, the sand–O_3_–BAC system had already been operated by 14 months more than O_3_–BAC–sand system. Such BAC condition may still retain some adsorption capacity as suggested [38] in a separate study. Separately, the specific degrading microbes in the BAC filter may have possibly developed during the filter operation, yielding additional treatment capacity. Some previous studies have shown that biodegradation plays an important role in pesticide removal in the BAC process [39].

## Conclusion

A comparison was conducted for the sand–O_3_–BAC and the O_3_–BAC–sand process in terms of their performance in the removal of particles, organic matter and micropollutants. On average, the results showed turbidity of 0.12 NTU in sand–O_3_–BAC vs. 0.06 NTU in O_3_–BAC–sand, and the average counts for particles of 2–5 µm, 5–15 µm and > 15 µm of O_3_–BAC–sand were 331.45, 50.06 and 3.14 count/mL, respectively, compared to 460.39, 109.80, and 14.02 count/mL, respectively, in sand–O_3_–BAC. However, the relocation of the sand filter might sacrifice efficiency in the removals of organic matter (by about 7%) and micropollutants. Thus, for the O_3_–BAC–sand application, some measures of enhanced coagulation should be suggested to improve the turbidity and particle removal, which would be helpful for enhancing the organic matter removal.

## Figures and Tables

**Table 1:** Information of the twenty-five pesticides compounds

No.	Compounds	CAS	Structure	Mol. wt.
1	Hexachlorobenzene (HCB)	118–74–1	C_6_Cl_6_	284.78
2	Heptachlor	76–44–8	C_10_H_5_C_l7_	373.35
3	1-Chloro-2-[2,2,2-trichloro-1-(4-chlorophenyl)ethyl] benzol	789–02–6	C_14_H_9_C_l5_	354.49
4	2,2-bis(p-Chlorophenyl)-1,1,1-trichloroethane	50–29–3	C_14_H_9_Cl_5_	354.49
5	2,2-bis(p-Chlorophenyl)-1,1-dichloroethane	72–54–8	C_14_H_10_Cl_4_	320.04
6	2,2-Bis(4-chlorophenyl)-1,1-dichloroethylene	72–55–9	C_14_H_8_Cl_4_	318.03
7	Lindane(r-BHC)	58–89–9	C_6_H_6_Cl_6_	290.83
8	α-Hexachlorocyclohexane (BHC)	319–84–6		290.82
9	β-Hexachlorocyclohexane (BHC)	319–85–7		290.83
10	δ-Hexachlorocyclohexane (BHC)	319–86–8		290.83
11	Dichlorvos	62–73–7	C_4_H_7_O_4_Cl_2_P	220.98
12	Malathion	121–75–5	C_10_H_19_O_6_PS_2_	330.35
13	Dimethoate	60–51–5	C_5_H_12_NO_3_PS_2_	229.25
14	Parathion	56–38–2	C_10_H_14_NO_5_PS	375
15	Parathion-methyl	298–00–0	C_8_H_10_NO_5_PS	263.2
16	Atrazine	1912–24–9	C_8_H_14_ClN_5_	215.68
17	Deltamethrin	52918–63–5	C_22_H_19_Br_2_NO_3_	505.2
18	Chlorothalonil	1897–45–6	C_8_Cl_4_N_2_	265.91
19	Chlorpyrifos	2921–88–2	C_9_H_11_Cl_3_NO_3_PS	350.59
20	Acetochlor	34256–82–1	C_14_H_20_ClNO_2_	269.77
21	Fenobucarb	3766–81–2	C_12_H_17_NO_2_	207.27
22	Butyl 2,4-dichlorophenoxyacetate	94–80–4	C_12_H_14_Cl_2_O_3_	277.15
23	Machette	23184–66–9	C_17_H_26_ClNO_2_	311.85
24	Dicofol	115–32–2	C_14_H_9_Cl_5_O	370.49
25	Monocrotophos	6923–22–4	C_7_H_14_NO_5_P	223.16

**Table 2:** COD_Mn_ of different fractions along the treatment processes (mg/L)

	RW	SE	SF	PO	BAC
Sand–O_3_–BAC					
TCOD	3.96	2.64	2.28	2.15	1.56
SCOD	2.34	2.05	1.93	1.81	1.54
InSCOD	1.61	0.59	0.35	0.34	0.02
O_3_–BAC–sand					
TCOD	3.98	2.57	1.66	2.37	1.72
SCOD	2.34	2.07	1.53	1.89	1.66
InSCOD	1.63	0.49	0.13	0.48	0.06

*RW* raw water, *SE* sedimentation, *SF* sand filtration, *PO* post-ozonation

**Table 3:** Contribution of total COD_Mn_ removal by each unit (%)

	SE	SF	PO	BAC	Total
Sand–O_3_–BAC	33.35	9.11	3.18	15.40	60.53
O_3_–BAC–sand	35.44	1.55	5.00	16.23	58.22

*SE* sedimentation, *SF* sand filtration, *PO* post-ozonation

**Table 4:** Concentration of the detected pesticides in each treatment process (ng/L)

	Machette	HCB	p,p′-DDT	Dimethoate	Fenobucarb	Dichlorvos	Acetochlor	Atrazine
Sand–O_3_–BAC								
RW	24.58	2.33	66.52	35.17	89.29	70.88	73.37	560.94
SE	22.88	1.78	0.00	30.19	78.96	64.82	67.82	544.09
SF	14.34	1.62	0.00	9.40	60.96	45.52	57.71	484.94
PO	14.13	1.78	0.00	8.65	31.01	36.88	50.80	352.08
BAC	10.52	0.71	0.00	6.43	17.01	25.66	34.05	104.71
O_3_–BAC–sand								
RW	36.68	1.78	6.54	17.08	21.38	53.09	162.76	542.23
SE	24.98	1.31	11.48	18.17	22.53	56.90	140.62	444.58
PO	21.39	1.46	5.96	12.59	10.80	53.73	119.76	416.54
BAC	11.69	1.17	0.00	5.13	8.75	48.00	67.45	141.70
SF	13.01	0.99	0.00	3.47	23.23	40.23	53.53	117.70

*RW* raw water, *SE* sedimentation, *SF* sand filtration, *PO* post-ozonation

## Abbreviations

O_3_–BAC–sand: O_3_–BAC with rear sand filtration
sand–O_3_–BAC: regular O_3_–BAC
DOC: dissolved organic carbon
COD_Mn_: chemical oxygen demand by KMnO_4_ titration
SCOD_Mn_: soluble COD_Mn_
inSCOD_Mn_: insoluble COD_Mn_
TCOD_Mn_: total COD_Mn_
GC/MS: gas chromatography and mass spectrometry
FPA: favor profle analysis
DMDS: dimethyl disulfide
2-MIB: 2-methylisoborneol
GC × GC–TOFMS: comprehensive two-dimensional gas chromatography with time-of-fight mass spectrometry
HCB: hexachlorobenzene
p,p′-DDT: 2,2-bis(p-Chlorophenyl)-1,1,1-trichloroethane
